# Implementation of a dispatch-instruction protocol for cardiopulmonary resuscitation according to various abnormal breathing patterns: a population-based study

**DOI:** 10.1186/s13049-015-0145-8

**Published:** 2015-09-17

**Authors:** Hidetada Fukushima, Masami Imanishi, Taku Iwami, Hironori Kitaoka, Hideki Asai, Tadahiko Seki, Yasuyuki Kawai, Kazunobu Norimoto, Yasuyuki Urisono, Kenji Nishio, Kazuo Okuchi

**Affiliations:** Department of Emergency and Critical Care Medicine, Nara Medical University, Shijo-cho, 840, Kashihara City, Nara 634-8522 Japan; Nara Saiseikai Gose Hospital, Mimuro, 20, Gose City, Nara 639-2306 Japan; Kyoto University Health Service, Yoshida-honmachi, sakyo-ku, Kyoto, 606-8501 Japan; Department of General Medicine, Nara Medical University, Shijo-cho, 840, Kashihara City, Nara 634-8522 Japan

## Abstract

**Background:**

We modified the dispatch protocol for cardiopulmonary resuscitation (CPR) using results of a retrospective analysis that identified descriptions by laypersons of possible patterns of agonal respiration. The purpose of this study was to assess the effectiveness of this modified protocol by comparing the frequency of dispatch instructions for CPR and bystander CPR before and after protocol implementation. We also identified descriptions of abnormal breathing patterns among ‘Not in cardiac arrest (CA)’ unresponsive cases.

**Methods:**

This study was conducted prospectively using the population-based registry of out-of-hospital cardiac arrests (OHCAs). For 8 months we implemented this modified protocol in cooperation with 4 fire departments that cover regions with a total population of 840,000.

**Results:**

There were 478 and 427 OHCAs before and after implementation, respectively. Among them, 69 and 71 layperson-witnessed OHCAs for pre- and post-implementation, respectively, were analyzed. Dispatchers provided CPR instructions more frequently after protocol implementation than before (55/71 [77.5 %] vs. 41/69 [59.4 %], p < 0.05). Based on breathing patterns described by emergency callers, dispatchers assessed 143 ‘Not in CA’ unresponsive cases and provided CPR instruction for 45 cases. Sensitivity and specificity of this protocol was 93 % and 50 %, respectively.

**Conclusions:**

This modified protocol based on abnormal breathing described by laypersons significantly increased CPR instructions. Considering high sensitivity and low specificity for abnormal breathing to identify CA and the low risk of chest compression for ‘Not in CA’ cases, our study suggested that dispatchers can provide CPR instruction assertively and safely for those unresponsive individuals with various abnormal breathing patterns.

## Background

Sudden cardiac arrest (CA) is a leading cause of deaths in the industrialized world. Despite the recent scientific progress in resuscitation, bystander CPR remains essential for survival of out-of-hospital CA (OHCA) without neurological deficits [1, 2]. CPR performed by laypersons who witness sudden CA can more than double the chance of survival from CA [3]. The incidence of bystander CPR, however, remains low with only 30 to 40 % of all OHCAs performed by bystanders [2, 4, 5]. Emergency medical service (EMS) dispatchers who take emergency calls play a key role in the performance of bystander CPR prior to the arrival of EMS personnel on the scene [4, 6]. EMS dispatch instructions for CPR can double the frequency of bystander CPR [7]. The identification of CA via telephone, however, is extremely difficult, especially when a collapsed individual has agonal respiration [8–10]. Laypersons often describe agonal respiration in various ways, such as snoring, wheezing, or weak breathing [8, 11, 12]. The current guidelines recommend EMS dispatchers to identify CA if a sudden collapsed individual is not responsive and not breathing normally. This protocol for identification of CA is called the ‘2-question protocol’. However, unresponsive persons who are not in CA also breathe abnormally [13]. The identification of CA presenting with agonal respiration by the 2-question protocol, therefore, would be difficult and the effectiveness or feasibility of the 2-question protocol has not been fully evaluated [9, 12]. For better implementation of this protocol and effective CA identification, EMS dispatchers should recognize how laypersons would describe agonal respiration.

In order to assist regional EMS dispatchers in identifying CA cases more correctly and in more frequently providing CPR instruction, we modified the regional dispatch protocol for CPR using descriptions of possible agonal respiration used by laypersons, which had been identified by our previous retrospective study [12]. The purpose of this study is to assess the effectiveness of this modified protocol by comparing the frequency of dispatch instructions for CPR and bystander CPR before and after the implementation of the protocol. We also examined descriptions of respiration for unresponsive patients without CA (‘Not in CA’ cases).

## Methods

This study was approved by the ethical committee of Nara Medical University.

### EMS system and dispatch protocol in Nara

**Fig. 1:**
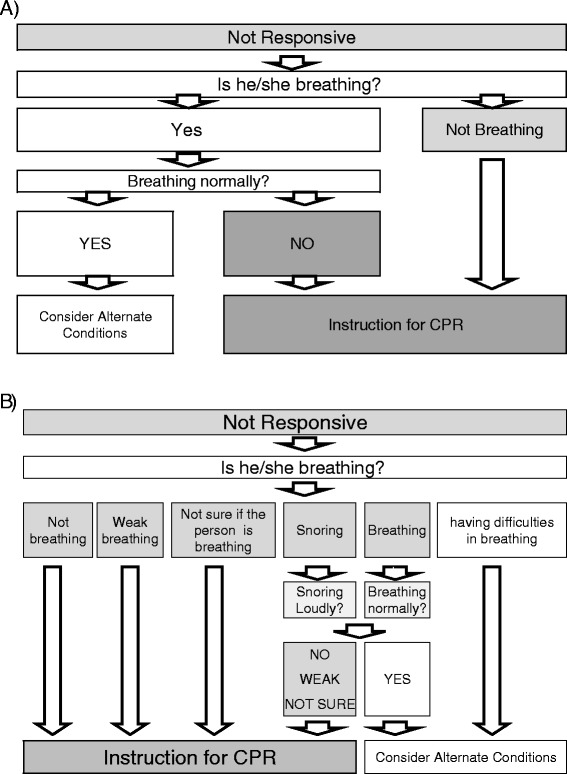
**a** Original regional emergency medical service (EMS) dispatch protocol for cardiac arrest. **b** Modified protocol for EMS dispatchers to identify cardiac arrest and provide cardiopulmonary resuscitation (CPR) instruction. If the unresponsive cases are described by laypersons as 1) not breathing, 2) breathing weakly, 3) snoring weakly or not normally, 4) not breathing normally, or 5) with breathing patterns that cannot be determined or described, EMS dispatchers are recommended to identify cases as cardiac arrest

The free emergency telephone number 119 is used to call for an ambulance in Japan. All EMS dispatchers are trained firefighters. Before this study, fire stations had regional dispatch protocols for CPR based on the 2-question protocol [6] (Fig. 1a). To identify CA, EMS dispatchers asked 119 callers whether the unresponsive case was breathing. If the answer was “yes,” EMS dispatchers asked whether the person was breathing normally and considered the possibility of agonal respiration. Once CA was suspected, the dispatchers instructed the caller to perform chest compression or conventional cardiopulmonary resuscitation. Dispatchers did not ask laypersons to check the pulse of the individual.

### Modified protocol for CA identification based on various breathing descriptions

We identified the key descriptions for identifying CA cases with possible agonal respiration in the retrospective analysis of 3-year reports of OHCA in Nara Prefecture (3700 km^2^ with 1.4 million population) [12]. Those descriptions were 1) not breathing, 2) weak breathing, 3) not sure if the person is breathing, 4) having difficulties in breathing, and 5) snoring. From these and based on the 2-question protocol, we developed the following 5 key descriptions: ‘not breathing’, ‘weak breathing’, ‘not sure if the person is breathing’, ‘weak snoring’, and ‘not breathing normally” (Fig. 1b).

### Study design

As a preliminary study, we implemented this protocol in 4 fire departments that cover regions with a total population of 840,000. The annual incidence of OHCA in this area is 70 per 100,000 inhabitants. We conducted this study prospectively for 8 months (August 1, 2011 to March 31, 2012). We included all unresponsive emergency patients aged 18 years or older who were subsequently transported to medical institutions. Exclusion criteria were cases of trauma, choking, and hanging by the neck and persons who collapsed after the emergency call. To focus on the association of various abnormal breathing patterns indicating possible agonal respiration described by laypersons, we also excluded cases for whom OHCA was not witnessed and whose condition was witnessed by health care providers. To evaluate the effectiveness of this modified protocol, we compared the number of EMS dispatch instructions for CPR and bystander CPR procedures between the 8-month period before implementation of the modified protocol and the 8-month period after implementation.

Data were prospectively collected by use of a form based on the Utstein style of reporting on guidelines for OHCA, including age, sex, origin of CA, location of CA, disabilities in daily living, EMS dispatcher-assisted CPR instructions, bystander-initiated CPR, first documented rhythm, time course of resuscitation, advanced airway management, intravenous fluids, and epinephrine, as well as prehospital return of spontaneous circulation, and survival discharge from hospital at 1 month with good neurological outcome (overall performance category 1 or 2 [14]) after the event. Both chest compression-only CPR and conventional CPR with rescue breathing were considered as layperson CPR. Rescue breathing without chest compression was classified as no CPR. Along with these data, we collected information on which breathing patterns were reported by laypersons according to this modified protocol.

### Statistics

Prior to this study, the average provision of dispatch instructions for CPR was 55 % in the regions examined. Expecting an average of 80 % dispatch instructions by this new study protocol with the condition of an alpha error of 5 % and a power of 80 %, we estimated a required sample size of 62 for each group.

Data were presented as medians and interquartile ranges for continuous variables and numbers and percentages for categorical variables. Groups were compared using the Mann-Whitney *U* test and chi-square test. All statistical analyses were two-sided and performed using computer software (SPSS ver. 22, Chicago, IL, USA). Results were considered to be statistically significant at a *P* value <0.05.

## Results

**Fig. 2:**
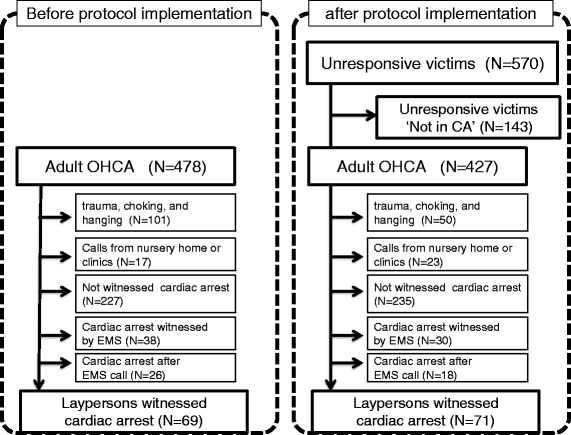
Study population. OHCA; out-of-hospital cardiac arrest, EMS; emergency medical service

After protocol implementation, dispatchers assessed 570 unresponsive cases (427 CA, 143 not CA). Of the 427 CAs, 71 layperson-witnessed CAs were analyzed. The control group was comprised of 69 cases of layperson-witnessed CA out of 478 CAs in the before protocol implementation period (Fig. 2). Table 1 shows the characteristics of CA cases before and after protocol implementation. The characteristics of both groups were similar except that the time interval between call receipt and hospital arrival in the before protocol implementation group was a little longer than in the after protocol implementation group (37 min vs. 36 min, p < 0.05).

**Table 1:** Baseline characteristics of the study groups

	After protocol implementation		Before protocol implementation		*p* value
Characteristics	(N = 71)		(N = 69)		
Male, n (%)	39	(54.9)	45	(65.2)	0.214
Age, y, median (IQR)	77	(67-86)	76	(68-83)	0.362
Cases with disabilities in daily life, n (%)	13	(18.3)	6	(8.7)	0.097
Cardiac arrest at home, n (%)	58	(81.7)	59	(85.5)	0.542
Cardiogenic cardiac arrest, n (%)	58	(81.7)	54	(78.3)	0.612
Initial rhythm of VF/VT, n (%)	8	(11.3)	15	(21.7)	0.095
Call - Arrival Time, min, median (IQR)	7	(6-10)	8	(6-10)	0.724
Advanced airway management by EMS, n (%)	40	(56.3)	48	(69.6)	0.101
Epinephrine administration by EMS, n (%)	47	(66.2)	46	(66.7)	0.953
Time from call to hospital arrival, min, median (IQR)	36	(28-42)	37*	(34-46)	0.044
Survival with good neurological outcome, n (%)	1	(1.4)	3	(4.3)	0.297

All continuous variables were presented as median and interquartile range and categorical variables were expressed as numbers and percentages. *IQR* interquartile range, *VF/VT* ventricular fibrillation and pulseless ventricular tachycardia, *EMS* emergency medical service, *CPR* cardiopulmonary resuscitation.*p < 0.05 by chi-square test

After implementation of this modified protocol, EMS dispatchers provided CPR instruction significantly more often than in the before protocol implementation group (55/71 [77.5 %] vs. 41/69 [59.4 %], p < 0.05). Bystander CPR, although statistically insignificant, was also provided more often in the after protocol implementation group than in the before protocol implementation group (44/71 [62.0 %] vs. 32/69 [46.4 %], p > 0.05, Table 2). During the after protocol implementation period, EMS dispatchers also provided CPR instruction for 45 non-CA cases (31.5 %). Among these cases, dispatch-assisted CPR was performed in 14 cases (31.1 %).

**Table 2:** Frequency of EMS dispatch-instructions for CPR and bystander CPR before and after protocol implementation

	Cardiac arrest	Cardiac arrest	*p* value
	After protocol implementation	Before protocol implementation	
	(N = 71)	(N = 69)	
EMS dispatched instruction for CPR, n (%)	55* (77.5)	41 (59.4)	0.021
Bystander CPR, n (%)	44 (62.0)	32 (46.4)	0.064

^*^Chi-square test

Breathing patterns in 71 CA cases and 143 of ‘Not in CA’ cases according to the modified protocol are shown in Table 3. The most frequent description for CA cases was ‘not breathing’ and for ‘Not in CA’ cases ‘breathing normally’ and ‘snoring normally’ were frequent descriptions. The frequency of other abnormal breathing patterns was similar between CA and ‘Not in CA’ cases. The sensitivity and specificity of this modified protocol to identify CA cases were retrospectively determined to be 93 % and 50 %, respectively.

**Table 3:** Breathing patterns of unresponsive cases with the modified protocol

	Laypersons wittnessed Cardiac arrest (N = 71)	Not in Cardiac arrest (N = 143)	*p* value
	n (%)	n (%)	
Not breathing	40 (56.3)	17 (11.7)	*P* < 0.001
Weak breathing	5 (7.0)	11 (7.6)	
Not sure whether the person is breathing or not	17 (23.9)	26 (17.9)	
Breathing difficulties		7 (4.8)	
Snoring weakly	1 (1.4)	4 (2.8)	
Not breathing normally	3 (4.2)	13 (9.0)	
Breathing normally	4 (5.6)	54 (37.2)	
Snoring normally	1 (1.4)	11 (7.6)	

The characteristics and diagnoses for ‘Not in CA’ cases are shown in Table 4. The median age was 78 years and 49.7 % were male. The most frequent diagnoses upon hospital arrival were syncope, conscious disturbance of unknown origin, stroke, and seizures.

**Table 4:** Characteristics of cases not in cardiac arrest

	Total (N = 143)
Age, y, mean, (IQR)	78 (60-84)
Male sex, n (%)	72 (50.3)
Assessment on hospital arrival, n (%)	
Syncope	37 (25.9)
Conscious disturbance of unknown origin	26 (18.2)
Stroke	21 (14.7)
Seizures	12 (8.4)
Cardiovascular event	12 (8.4)
Hypoxia due to respiratory event	11 (7.7)
Other illness	7 (4.9)
Hypo/Hyperglycemia	7 (4.9)
Psychosis	6 (4.2)
Acute intoxication	4 (2.8)

## Discussion

In this prospective study, the implementation of a protocol modified with descriptions of possible agonal respiration by laypersons effectively increased EMS- dispatched instructions for CPR.

The main feature of this protocol was the modification of the 2-question protocol according to results of a population-based retrospective study of how laypersons describe possible agonal respiration. The most frequent description in CA cases was ‘not breathing’. Other breathing patterns that strongly indicate CA could not be determined in this current study. EMS dispatchers, however, did provide CPR instructions more often than in the before protocol implementation period. It is difficult to tell what actually helped dispatchers identify CA and provide CPR instruction more often than previously. We speculate that the list of abnormal breathing patterns possibly indicating agonal respiration might have helped dispatchers consider CA more frequently. Several studies have reported that only education on agonal respiration for EMS dispatchers can improve dispatch-identification of CA [15, 16].

In this current study, our modified protocol had high sensitivity of 93 % with lower specificity of 50 %. It was reported previously that the sensitivity and specificity of recommended dispatch protocols for CPR instruction ranged from 38 to 90 % and 95 to 99 %, respectively [17–22]. Since this study included layperson-witnessed CA cases, the sensitivity of this modified protocol was sufficiently high when compared with previous reports. Specificity, however, was much lower. Although the dispatch protocols in these previous reports were mostly based on the 2-question protocol, each had variations including the Medical Priority Dispatch System (MPDS). Furthermore, only 2 studies reported the specificity of protocols based on 2 questions [17, 18]. Specificity for identification of abnormal breathing among unresponsive cases with CA needs further evaluation. A recent population-based study by Tanaka et al. evaluating an EMS dispatch CPR instruction protocol reported that specificity of the 2-question protocol was 99.6 % [23]. This specificity, however, was calculated from all of the emergency cases transported by EMS. If the calculation would have been done exclusively on unresponsive cases at the time of emergency calls, this high specificity should have decreased.

A specific abnormal breathing pattern that is strongly related to CA has not been identified yet. In this study, the most frequently reported breathing pattern of CA cases was ‘not breathing’ (40/71 [56.3 %]). Other breathing patterns, however, had no association with CA identification. These findings are identical with a previous report by Berdowski et al. [10]. Berdowski et al. also noted several descriptions by laypersons such as facial color, no pulse, or even simply ‘he/she is dead’ as phrases that can help EMS dispatchers identify CA [10]. Recently, Tanaka et al. described the usefulness of additional information that possibly indicated CA [23]. Unfortunately, since we conducted this study focusing on the association between laypersons’ description of possible agonal respiration and dispatch instructions for CPR, no such additional information was available.

Dispatchers might be concerned about unnecessary CPR instructions and risks of CPR for ‘Not in CA’ cases. Several reports indicate risks of CPR. A systematic review and pooled analysis done by Miller et al. revealed that the incidence of CPR-associated major thoracic injuries such as aortic laceration, cardiac injury, pneumo/hemothorax or liver injury occur in up to 7 % of CA victims [24]. When it comes to unresponsive victims not in CA, however, the risk of CPR was extremely low. Previous studies reported that chest compression for those not in CA is much less hazardous resulting in chest discomfort or minor rib fractures [25, 26]. In this current study, dispatch-assisted CPR was performed in 14 ‘Not in CA’ cases. Although the number was small, we also did not receive any adverse reports from the doctors who were in charge of transported unresponsive cases who had undergone chest compression. Our study along with previous reports indicated that the risk of CPR to those not in CA is very low. Considering the high sensitivity and low specificity for abnormal breathing and low risk of chest compression for unresponsive persons not in CA, our study strongly suggested that EMS dispatchers can provide CPR instruction assertively and safely for those unresponsive cases with various abnormal breathing patterns described by laypersons.

This study has substantial limitations. As a preliminary study, we implemented this modified study protocol in small areas with a total population of 840,000. As a result, the numbers of study subjects were relatively small, so we were not able to analyze the association between this study protocol and the outcome in sudden CA cases. Additionally, dispatch instructions for CPR should be provided immediately. We were regretfully unable to assess the time course of CPR instruction according to this study protocol due to policies of each fire department regarding recorded emergency calls.

## Conclusions

The modified protocol based on the descriptions of possible agonal respiration by laypersons effectively improved EMS dispatch instructions for CPR. Failing to determine the key breathing patterns that indicate CA except for ‘not breathing’ resulted in sensitivity of 93 % and specificity of 50 % for this modified protocol. Considering the high sensitivity and low specificity of abnormal breathing for identifying CA and low risk of chest compression for ‘Not in CA’ cases, our results suggest that EMS dispatchers can provide CPR instruction assertively and safely for those unresponsive victims with various abnormal breathing patterns.

## Abbreviations

CA: Cardiac arrest
CPR: Cardiopulmonary resuscitation
EMS: Emergency medical service
OHCA: Out-of-hospital cardiac arrest

## References

[1] Iwami T, Nichol G, Hiraide A, Hayashi Y, Nishiuchi T, Kajino K (2009). Continuous improvements in “chain of survival” increased survival after out-of-hospital cardiac arrests: a large-scale population-based study. Circulation.

[2] Wissenberg M, Lippert FK, Folke F, Weeke P, Hansen CM, Christensen EF (2013). Association of national initiatives to improve cardiac arrest management with rates of bystander intervention and patient survival after out-of-hospital cardiac arrest. JAMA.

[3] Sasson C, Rogers MA, Dahl J, Kellermann AL (2010). Predictors of survival from out-of-hospital cardiac arrest: a systematic review and meta-analysis. Circ Cardiovasc Qual Outcomes.

[4] Song KJ, Shin SD, Park CB, Kim JY, Kim do K, Kim CH (2014). Dispatcher-assisted bystander cardiopulmonary resuscitation in a metropolitan city: A before-after population-based study. Resuscitation.

[5] Nichol G, Thomas E, Callaway CW, Hedges J, Powell JL, Aufderheide TP (2008). Regional variation in out-of-hospital cardiac arrest incidence and outcome. JAMA.

[6] Lerner EB, Rea TD, Bobrow BJ, Acker JE, Berg RA, Brooks SC (2012). Emergency medical service dispatch cardiopulmonary resuscitation prearrival instructions to improve survival from out-of-hospital cardiac arrest: a scientific statement from the American heart association. Circulation.

[7] Rea TD, Eisenberg MS, Becker LJ, Murray JA, Hearne T (2003). Temporal trends in sudden cardiac arrest: a 25-year emergency medical services perspective. Circulation.

[8] Bang A, Herlitz J, Martinell S (2003). Interaction between emergency medical dispatcher and caller in suspected out-of-hospital cardiac arrest calls with focus on agonal breathing. A review of 100 tape recordings of true cardiac arrest cases. Resuscitation.

[9] Bohm K, Rosenqvist M, Hollenberg J, Biber B, Engerstrom L, Svensson L (2007). Dispatcher-assisted telephone-guided cardiopulmonary resuscitation: an underused lifesaving system. Eur J Emerg Med.

[10] Berdowski J, Beekhuis F, Zwinderman AH, Tijssen JG, Koster RW (2009). Importance of the first link: description and recognition of an out-of-hospital cardiac arrest in an emergency call. Circulation.

[11] Clark JJ, Larsen MP, Culley LL, Graves JR, Eisenberg MS (1992). Incidence of agonal respirations in sudden cardiac arrest. Ann Emerg Med.

[12] Fukushima H, Imanishi M, Iwami T, Seki T, Kawai Y, Norimoto K (2015). Abnormal breathing of sudden cardiac arrest victims described by laypersons and its association with emergency medical service dispatcher-assisted cardiopulmonary resuscitation instruction. Emerg Med J.

[13] Bang A, Gustavsson M, Larsson C, Holmberg S, Herlitz J (2008). Are patients who are found deeply unconscious, without having suffered a cardiac arrest, always breathing normally?. Resuscitation.

[14] Cummins RO, Chamberlain DA, Abramson NS, Allen M, Baskett P, Becker L (1991). Recommended guidelines for uniform reporting of data from out-of-hospital cardiac arrest: the Utstein Style. Task Force of the American Heart Association, the European Resuscitation Council, the Heart and Stroke Foundation of Canada, and the Australian Resuscitation Council. Ann Emerg Med.

[15] Perkins GD, Walker G, Christensen K, Hulme J, Monsieurs KG (2006). Teaching recognition of agonal breathing improves accuracy of diagnosing cardiac arrest. Resuscitation.

[16] Bohm K, Stalhandske B, Rosenqvist M, Ulfvarson J, Hollenberg J, Svensson L (2009). Tuition of emergency medical dispatchers in the recognition of agonal respiration increases the use of telephone assisted CPR. Resuscitation.

[17] Clark JJ, Culley L, Eisenberg M, Henwood DK (1994). Accuracy of determining cardiac arrest by emergency medical dispatchers. Ann Emerg Med.

[18] Flynn J, Archer F, Morgans A (2006). Sensitivity and specificity of the medical priority dispatch system in detecting cardiac arrest emergency calls in Melbourne. Prehosp Disaster Med.

[19] Eisenberg MS, Hallstrom AP, Carter WB, Cummins RO, Bergner L, Pierce J (1985). Emergency CPR instruction via telephone. Am J Public Health.

[20] Garza AG, Gratton MC, Chen JJ, Carlson B (2003). The accuracy of predicting cardiac arrest by emergency medical services dispatchers: the calling party effect. Acad Emerg Med.

[21] Kuisma M, Boyd J, Vayrynen T, Repo J, Nousila-Wiik M, Holmstrom P (2005). Emergency call processing and survival from out-of-hospital ventricular fibrillation. Resuscitation.

[22] Ma MH, Lu TC, Ng JC, Lin CH, Chiang WC, Ko PC (2007). Evaluation of emergency medical dispatch in out-of-hospital cardiac arrest in Taipei. Resuscitation.

[23] Tanaka Y, Nishi T, Takase K, Yoshita Y, Wato Y, Taniguchi J, et al. Survey of a protocol to increase appropriate implementation of dispatcher-assisted cardiopulmonary resuscitation for out-of-hospital cardiac arrest. Circulation. 2014;129:1751–60. 10.1161/CIRCULATIONAHA.113.004409 24508824

[24] Miller AC, Rosati SF, Suffredini AF, Schrump DS. A systematic review and pooled analysis of CPR-associated cardiovascular and thoracic injuries. Resuscitation. 2014;85:724–31. 10.1016/j.resuscitation.2014.01.028 PMC4031922 24525116

[25] White L, Rogers J, Bloomingdale M, Fahrenbruch C, Culley L, Subido C (2010). Dispatcher-assisted cardiopulmonary resuscitation: risks for patients not in cardiac arrest. Circulation.

[26] Haley KB, Lerner EB, Pirrallo RG, Croft H, Johnson A, Uihlein M (2011). The frequency and consequences of cardiopulmonary resuscitation performed by bystanders on patients who are not in cardiac arrest. Prehosp Emerg Care.

